# Purification of the Thames

**Published:** 1859-01

**Authors:** 


### MISCELLANEA.

### PURIFICATION OF THE THAMES.

The subjoined is the substance of a letter addressed by Mr. F. O. Ward to William Coningham, Esq., M.P., and printed by Mr. Coningham for circulation:—

"In the first place I would remind you, that to throw away the ammonia and phosphorus of the London sewage is virtually to throw away bread. Town sewage, which many engineers look upon as refuse to be discharged, I regard as property to be administered. The proper outfall for the London sewage is not this or that point of the river or of the sea, but a suitable tract of land growing exhausting crops. Fifty farms of a thousand acres each might be raised in value, at least ten pounds per acre per annum, by irrigation with the London sewage. This would produce five hundred thousand pounds per annum, equivalent at five per cent. to ten millions of capital. This ought not to be thrown into the sea.

"In the next place I would point out that just as, on the one hand, the sewage proper should be carefully diverted *from* the Thames, just so, on the other hand, should the rainfall be carefully directed *to* the Thames, to aid its scour; which suffers by every drop withdrawn. To divert a rain brook is to mutilate a river. And every extension of an intercepting system having the amputation of tributaries for its object and effect, must progressively impoverish the main stream. So that, were the Thames to be 'purified' on this plan from end to end, it would be pruned of all its branches; and its whole fresh water stream would be diverted into the intercepting tunnels. The tidal estuary would remain, but the *river* would cease to exist. Its channel would be free from sewage no doubt, but it would also be free from water; and the perfection of purity would correspond to the moment of abolition. This is not what we want; we want a river running pure, but also flowing full.

"Hence, it appears that sewage and rainfall, though valuable when separate, the one to fertilize land, the other to scour streams, are rendered worthless by admixture. Each spoils the other—sewage rainfall by pollution—rainfall sewage by dilution. The mixed mass is too vast and variable for economical distribution over fields, too foul and fœtid for advantageous delivery down streams.

"On this point it is worth while to dwell, for it is the key of the problem.

"The London sewage proper consists of the (say) fifty million gallons which the water companies pump daily into the town, enriched with the residuary matter which this water takes up in its passage through the dwellings of the population. The average weight of residuum (excluding moisture), yielded to the sewage by each man, woman, and child, is two ounces per diem; or, for the two and a half million inhabitants of London, 139 tons per diem, containing, at seventeen per cent., twenty-three tons and a half of nitrogen, equal to twenty-eight tons and a half of ammonia: which ammonia, at sixpence per pound, is worth £1597. The weight of the pipe-water thus enriched—in other words, of the daily sewage proper, is, in round numbers, only 223 thousand tons; about as much as a heavy shower of rain throws down on two thousand acres of land. This small and uniform daily sewage flow would require only a couple of moderate sized sewers (instead of several colossal tunnels), to convey it away; and, if necessary it could be pumped this way or that by steam power, as easily as a lady pours tea into this or that cup at her pleasure.

"Turn now to the rainfall, and consider the contrast it presents, in this respect, to the sewage.

"The rainfall on the London drainage area, taking this at only fifty-nine and a half square miles, and making ample allowance for evaporation and absorption, may yield to the sewage some eighty or ninety million tons annually; a total which may be taken as about equal to the annual total of sewage. If, therefore, the rain drizzled down uniformly all the year through, so as to afford, like the sewage, a regular daily supply, it could be mastered, like sewage, without difficulty. This, however, as we all know, is not the case. The whole of the rain falls on 152 days of the year; and of the annual twenty-four inches, sixteen fall in forty-four days—or two-thirds of the rain in about one one-eighth of the days. On one day in twelve throughout the year the rainfall is to the sewage of London as four and three quarters to one; on a smaller number of days (about ten in each year), the proportion of rainfall to sewage is as nine and a half to one; and on some few occasions annually it is as nineteen to one and upwards. This disproportion is rendered still greater by the fact that the rainfall assigned to each rainday is not really diffused over twenty-four hours of time, but nearly always descends on a fractional portion thereof; so that, for example, seven million tons of rain, equal to more than a month's sewage, sometimes fall on London in a single hour. The mixed streams of rainfall and sewage, liable to be thus suddenly swollen, exceed the capacity of any tunnels that can be built for their diversion from the river; and would overpower any mechanism at our disposal for their distribution upon the soil. The great brook sewers already existing, and the great rain and sewage tunnels which it is proposed to build for their interception, are equally open to this objection—that their current, on rainy days torrential, must needs shrink in dry weather to a slender streamlet, too weak to scour the containing culvert so as to prevent the accumulation of putrescent deposit.

"Reflect now, for a moment, how this fact of accumulation, of stagnancy instead of circulation, changes all the conditions of the problem. The daily dry fœcal discharge from London amounts only, as we have already seen, to 139 tons; a comparatively insignificant quantity if delivered as fast as produced. But instead of taking measures to secure for London this regular diurnal evacuation, we keep, on the most moderate estimate, at least twelve months *excreta* constantly stagnating underground as deposit in the cesspools and sewers. The mass of putridity thus constantly retained in subterranean London actually equals one day's evacuation of the whole population of Europe and Asia, numbering eight hundred millions. The figure is a startling one, and the fact still more so ; but a simple calculation proves it true : for the number of the London population, multiplied by 365, gives a product exceeding eight hundred millions.

"Now consider the effect of a sudden rain-storm falling on London, and pouring through these overcharged subterranean receptacles. Suppose it only to sweep into the river nine or ten day's accumulation of filth, to what do you imagine that is equivalent? It is equivalent to the simultaneous discharge into the river Thames of the mass of excrement produced in one day by the entire population of Great Britain, numbering twenty-one millions. And to such eruptions of filth we should still be frequently liable, even if the great tunnels for mixed rainfall and sewage were built. The money loss on every such occasion would be, in ammonia only without reckoning phosphorus, nearly sixteen thousand pounds. Besides, after every such discharge, the tidal river would remain discoloured, and in hot weather, putrescent, for several days. Such would be the operation of the colossal tunnels on which we are invited to lay out millions; such are the evils consequent on the mingling of rainfall with sewage.

"The obvious conclusion is, that the tunnel scheme propounded by the Metropolitan Board of Works, with the sanction of Mr. Stephenson and his party, not merely ignores one half of the problem in hand, viz., the agricultural utilisation of the sewage, but is inadequate to accomplish the moiety which alone it contemplates, viz., the purification of the river.

"Indeed, by their last vote on this subject, the Metropolitan Board resolved to pour the sewage of the western district of London, more or less deodorised, into the Thames above Westminster Bridge, and to reserve the proposed great tunnels for the conveyance of the remaining sewage only. Of deodorisation, the mainstay of this scheme, I will only here remark that, while at best it is a costly and imperfect palliative it becomes quite impracticable precisely when most needed, *i.e.*, when heavy showers are sweeping from the sewers the largest masses of putrescent filth. Independently, however, of this objection, the two parts of the scheme are manifestly inconsistent. For, if deodorisation suffices for the west, why is interception necessary for the east? And, contrariwise, if miles of tunnel are required to convey far off the eastern sewage, how can it be right to pour the western sewage into the river above bridge? Surely the principle must be false that leads to such illogical conclusions.

"The principle to which, by these and other considerations, my friends and I have been gradually led, is shortly this—that *the whole of the rainfall is due to the river, the whole of the sewage to the soil.*

"The adoption of this principle is, we believe, as essential for the perfect purification of the Thames, as it is for the economical utilisation of the sewage.

"That this may be obvious to you, pray keep in view that when sewage and rainfall are once mixed, whether in the Thames itself or in the minutest of the filaments that feed it (in a street-sewer or in a house-drain for example), those mingled waters can never again be separated. In polluting the smallest of its tributaries you virtually pollute the Thames; and, as it has been said, 'Take care of the pence, and the pounds will take care of themselves," so I venture to say, "Purify the tributaries, and the main stream will run pure of itself."

"If, now, we trace in each house the course of the rain from roof and area, and the course of the sewage from closet and sink, till we come to the point at which the separate pipes conveying these two distinct streams meet in a single drain, we arrive at the precise boundary line between possible and impossible in this matter of Thames purification and sewage utilization. For, up to this point, and before this meeting of two waters, we are free to apply each streamlet to its proper use. We can send the unpolluted rainfall to scour the river, and the undiluted sewage to fertilise the land. But directly this junction point is passed, directly the daily runlet of cistern-water, rich with its freight of ammonia and phosphorus, meets and mingles with the casual rainfall, the two waters become, as we have seen, a worthless unmanageable mixture, equally unfit for agricultural and urban use. Not only do they cease to be our property, and pass beyond the control of art, but they revert to the domain of nature, spoiled even for her simple service. For this error we are punished by pestilence.

"I say, therefore, that the battle of Interception is to be fought, not on the banks of the river, but in the basements of the houses; not with monstrous tunnels, but with modest tubes; not by the diversion of variable rain-brooks, alternately dry and torrential, but by the diversion of uniform cistern supplies, always moderate and manageable; not at a profitless cost of many millions, yielding no return, but at a profitable outlay of few millions, producing an ample return,—probably half a million per annum.

"This tubular purification of rivers, and fertilisation of lands, is indeed but the logical extension of the tubular drainage of houses and streets, which my friends and I have succeeded in establishing after a ten years' struggle with the engineers. And as our tubular sewers, notwithstanding the strenuous opposition of Mr. Stephenson and his friends, are now working successfully by hundreds of miles, not only in provincial towns, but in the metropolis itself: so also, I am confident, will the tubular purification of the Thames ultimately supersede the monstrous tunnel project, which, if adopted, would cost us many millions, and turn out a gigantic failure after all.

"You will observe that, in this short note, I have confined myself to the summary indication of a few broad principles; abstaining purposely from the premature development of a specific plan for their realisation. My name has indeed been attached without my sanction, to plans and calculations which have been widely circulated, ostensibly to illustrate, but really to discredit, the principles which I advocate. It has even been asserted that the realisation of those principles would involve the tearing up of every basement in London, the re-construction in duplicate of the drains from back to front of every house, and from end to end of every street,—with other equally preposterous extravagancies. Such conclusions are not mine, and they argue only poverty of invention on the part of those who can imagine no simpler means of carrying my views into effect.

"I have only to add, in conclusion, that the purification of rivers and the utilisation of sewage are, in my judgment, but two aspects or incidents of a sanitary organisation, comprising several other elements, each indispensable to the perfect working of the whole. This complete organisation cannot, however, be suddenly accomplished; nor can even its several parts be simultaneously prepared. But in the development of such portions as we may be able presently to undertake, the others may be kept in view. And this is in the highest degree desirable, in order that the sanitary works of our day may operate, not as a bar, but as a transition, to the more perfect institutions of our successors. Should the monstrous rain-and-sewage tunnels, proposed by the Metropolitan Board, be built, they would indeed oppose a serious obstacle to such ulterior progress. But of this I have little fear. Those subterranean rivers are already beginning to be regarded by the ratepayers as a costly and colossal blunder; and, unless I am much mistaken, they will be obsolete before they are begun."

We have reprinted the whole of Mr. Ward's letter under the impression that the views it expresses cannot be too strongly enforced. The scheme which he so ably denounces is now (somewhat modified in character, certainly, but not less objectionable) too far arranged to be recalled, but this is no reason why the gigantic blunder about to be carried out should be left uncriticised. We concur with Mr. Ward, that the monstrous error will be discovered before it is finished, but not, we fear, before a few millions of money have been expended on an absurd experiment. [EDITOR.]

